# The structure of alanine racemase from *Acinetobacter baumannii*

**DOI:** 10.1107/S2053230X14017725

**Published:** 2014-08-29

**Authors:** Emily Davis, Emma Scaletti-Hutchinson, Helen Opel-Reading, Yoshio Nakatani, Kurt L. Krause

**Affiliations:** a Department of Biochemistry, University of Otago, Dunedin, New Zealand

**Keywords:** *Acinetobacter baumannii*, alanine racemase

## Abstract

*Acinetobacter baumannii* is an opportunistic Gram-negative bacterium which is a common cause of hospital-acquired infections. Numerous antibiotic-resistant strains exist, emphasizing the need for the development of new antimicrobials. Alanine racemase (Alr) is a pyridoxal 5′-phosphate dependent enzyme that is responsible for racemization between enantiomers of alanine. As d-alanine is an essential component of the bacterial cell wall, its inhibition is lethal to prokaryotes, making it an excellent antibiotic drug target. The crystal structure of *A. baumannii* alanine racemase (Alr*_Aba_*) from the highly antibiotic-resistant NCTC13302 strain has been solved to 1.9 Å resolution. Comparison of Alr*_Aba_* with alanine racemases from closely related bacteria demonstrates a conserved overall fold. The substrate entryway and active site of the enzymes were shown to be highly conserved. The structure of Alr*_Aba_* will provide the template required for future structure-based drug-design studies.

## Abstract 2

The crystal structure of *A. baumannii* alanine racemase (Alr*_Aba_*) from the highly antibiotic resistant NCTC13302 strain has been solved to 1.9 Å resolution. Comparison of (Alr*_Aba_*) with alanine racemases from closely related bacteria demonstrates a conserved overall fold.

## 1. Introduction   

*Acinetobacter baumannii* is a small nonmotile Gram-negative bacterium that is capable of replicating under a wide range of environmental conditions (Peleg *et al.*, 2008 ▶). In vulnerable patients, *A. baumannii* can cause severe infections such as pneumonia, septicaemia, urinary-tract infections and meningitis (Lee *et al.*, 2007 ▶). There are numerous strains of *A. baumannii* that are responsible for endemics and epidemics in hospitals (Scott *et al.*, 2007 ▶; Eliopoulos *et al.*, 2008 ▶) and many of these strains are antibiotic resistant (Zavascki *et al.*, 2010 ▶). In fact, multidrug-resistant *A. baumannii* is considered to be among the most difficult Gram-negative bacteria to control and treat (Dijkshoorn *et al.*, 2007 ▶). Therefore, new drug therapies are vital in combating these infections (Joshi *et al.*, 2003 ▶; Gootz & Marra, 2008 ▶).

Alanine racemase (EC 5.1.1.1) is a pyridoxal 5′-phosphate (PLP)-dependent enzyme which catalyses racemization between l-alanine and d-alanine (Walsh, 1989 ▶). d-Alanine is an essential building block of peptidoglycan, a critical component of the bacterial cell wall (Hols *et al.*, 1997 ▶). Alanine racemase (Alr) is the sole source of d-alanine in many bacteria and its inhibition is lethal, making the enzyme an attractive antibiotic drug target (Lambert & Neuhaus, 1972 ▶). Kinetic studies of Alr have indicated that racemization occurs *via* a stepwise two-base mechanism, in which a highly conserved lysine and a tyrosine residue act as acid and base catalysts. These residues either accept or donate the α-hydrogen of the alanine substrate, depending on the direction of the reaction (Watanabe *et al.*, 2002 ▶; Spies & Toney, 2003 ▶).

Alanine racemase has been structurally characterized from numerous bacteria, including *Geobacillus* (formerly *Bacillus*) *stearo­thermophilus* (Shaw *et al.*, 1997 ▶), *Pseudomonas aeruginosa* (LeMagueres *et al.*, 2003 ▶), *Streptomyces lavendulae* (Noda *et al.*, 2004 ▶), *Mycobacterium tuberculosis* (LeMagueres *et al.*, 2005 ▶), *Escherichia coli* (Wu *et al.*, 2008 ▶), *Bacillus anthracis* (Couñago *et al.*, 2009 ▶), *Enterococcus faecalis* (Priyadarshi *et al.*, 2009 ▶), *Bartonella henselae* (Abendroth *et al.*, 2011 ▶), *Streptococcus pneumoniae* (Im *et al.*, 2011 ▶), *Staphylococcus aureus* (Scaletti *et al.*, 2012 ▶) and *Oenococcus oeni* (Palani *et al.*, 2013 ▶). In these structures, the enzyme is a homodimer in which the two monomers interact in a head-to-tail fashion, generating two active sites. Each Alr monomer has two distinct domains: an α/β-barrel at the N-terminus and a predominantly β-stranded domain at the C-terminus. The PLP cofactor forms an internal aldimine linkage with the highly conserved catalytic lysine residue in the α/β-barrel domain (Shaw *et al.*, 1997 ▶). Here, we report the structure and partial kinetic characterization of *A. baumannii* alanine racemase (Alr*_Aba_*) from the multidrug-resistant NCTC13302 strain. A crystallization report concerning alanine racemase from this bacterium has appeared in this journal (Nguyen *et al.*, 2013 ▶), but no structure is available and it was not consulted for this work. The structure presented here will provide a template for future structure-based drug-design studies targeting this important enzyme.

## 2. Methods   

### 2.1. Gene cloning, expression and protein purification   

Prior to cloning, genomic DNA was extracted from *A. baumannii* strain NCTC13302, after which the alanine racemase *alr* gene was amplified by PCR using the following primers: 5′-ATGATCTACATATGCGTCAAGCAACAGTT-3′ (forward) and 5′-TATCTCGAG­TTAAGTACCCTGACGGAC-3′ (reverse). Full-length *A. baumannii alr* (residues 1–356) was cloned into pET-26b, which was transformed into *E. coli* BL21 (DE3) cells. Cells grown overnight at 310 K were used to inoculate the main culture, which was induced with 0.5 m*M* IPTG at an OD_600_ of 0.5. After expression for 16 h, the cell pellet was collected by centrifugation and the cells were lysed *via* sonication. Following ammonium sulfate cuts of 20 and 60%, the pellet fraction was resuspended in 20 m*M* Tris pH 8.0 and further purified by hydrophobic interaction, anion-exchange and size-exclusion chromatography. Fractions containing Alr*_Aba_* were greater than 95% pure as indicated by SDS–PAGE.

### 2.2. Crystallization   

Purified Alr*_Aba_* was concentrated to 6 mg ml^−1^ using a Vivaspin 20 (10 000 Da molecular-weight cutoff; GE Healthcare) and sitting drops were set up *versus* 100 m*M* 2-(*N*-morpholino)ethanesulfonic acid (MES) pH 6.0, 200 m*M* CaCl_2_, 20% polyethylene glycol 6000 (PEG 6000). Single yellow crystals grew within 4 d and were cryoprotected by soaking in mother liquor containing 20% glycerol. Crystals were flash-cooled in liquid nitrogen prior to data collection.

### 2.3. Data collection and processing   

A native Alr*_Aba_* data set was collected at the Australian Synchrotron (Melbourne, Australia) on the MX1 beamline equipped with an ADSC Quantum 315r image-plate detector. Data were collected at 13.000 keV using an oscillation angle of 0.5° and an exposure time of 2 s per image. Diffraction images were processed with *iMosflm* (Battye *et al.*, 2011 ▶), *POINTLESS* (Grosse-Kunstleve *et al.*, 2002 ▶) and *SCALA* (Evans, 2006 ▶) within the *CCP*4 suite (Winn *et al.*, 2011 ▶). The crystals of Alr*_Aba_* belonged to the monoclinic space group *P*2_1_, with unit-cell parameters *a* = 47.0, *b* = 83.0, *c* = 93.3 Å, α = 90, β = 97.1, γ = 90°. Data were collected and processed to 1.9 Å resolution with statistics as presented in Table 1 ▶.

### 2.4. Structure determination and refinement   

The structure of Alr*_Aba_* was solved *via* molecular replacement with *Phaser* (McCoy *et al.*, 2007 ▶) using the monomer of *P. aeruginosa* alanine racemase (with ligands and waters removed), to which it has a sequence identity of 41%, as the search model. This was performed assuming the presence of two monomers in the asymmetric unit, as suggested by the Matthews coefficient *V*_M_ of 2.07 Å^3^ Da^−1^ (Matthews, 1968 ▶). *PHENIX* (Adams *et al.*, 2010 ▶) was used to build the initial model and to change the amino-acid sequence to that of Alr*_Aba_*. After several rounds of model building and refinement using *Coot* (Emsley & Cowtan, 2004 ▶) and refinement using *REFMAC*5 (Murshudov *et al.*, 2011 ▶), the electron density improved and waters and the cofactor PLP were built into the structure. The final structure had an *R* factor of 19.7% and an *R*_free_ of 23.4%. The r.m.s. deviations of bond lengths and angles were 0.016 Å and 1.75°, respectively. Intermonomer interactions in the structure were analysed using the *Protein Interfaces, Surfaces and Assemblies* service (*PISA*) at the European Bioinformatics Institute (http://www.ebi.ac.uk/pdbe/prot_int/pistart.html; Krissinel & Henrick, 2007 ▶). Comparisons to other structures were made with the respective PDB files as deposited. The final model was validated using *PROCHECK* (Laskowski *et al.*, 1993 ▶), with the resulting Ramachandran plot indicating that 98.2% of the residues were in the most favoured regions, with 1.6% in additionally allowed regions. Additional structure-determination and refinement statistics are presented in Table 1 ▶.

### 2.5. Enzyme kinetics   

The d-alanine to l-alanine direction of the racemization catalysed by Alr*_Aba_* was characterized using a coupled-enzyme assay based on Esaki & Walsh (1986 ▶) and as described in previous studies (Strych *et al.*, 2000 ▶, 2001 ▶). This assay was carried out at 30°C and begins with the conversion of d-alanine to l-alanine by alanine racemase. The l-alanine is then converted to pyruvate and ammonia by l-alanine dehydrogenase, producing NADH, which is tracked by following the increase in absorbance at 340 nm. Concentrations of d-alanine ranging from 0.1 to 10 m*M* were assayed in triplicate using 200 ng Alr*_Aba_* per reaction. The production of NADH was monitored for 10 min with the slope calculated during the last 2 min of the assay. The kinetic constants *K*_m_ and *V*_max_ were determined using nonlinear regression fitting carried out within *GraphPad Prism* v.6 (GraphPad Software, La Jolla, California, USA).

## 3. Results and discussion   

### 3.1. Overall structure of *A. baumannii* alanine racemase   

**Figure 1:**
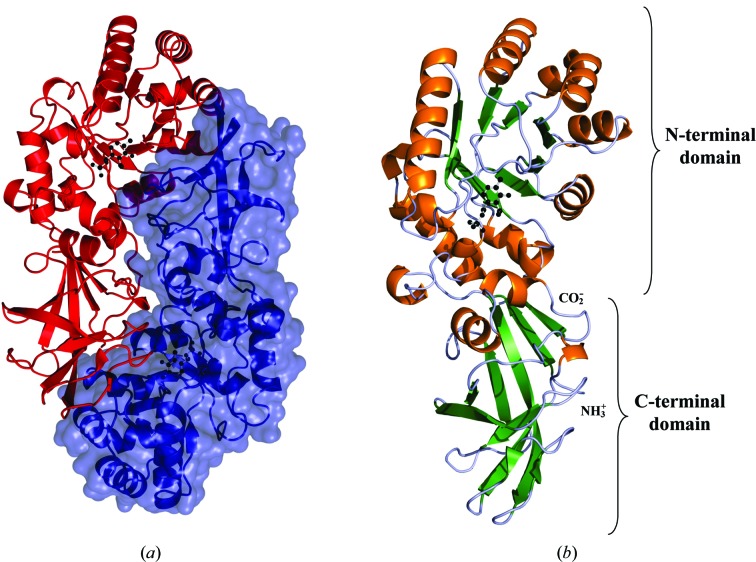
(*a*) Ribbon representation of the *A. baumannii* alanine racemase dimer. Monomers are coloured blue and red, with the surface representation of one monomer also shown in blue. The PLP cofactors are depicted as black ball-and-stick models. (*b*) Structure of the *A. baumannii* alanine racemase monomer. Ribbon representation with α-helices coloured orange and β-sheets shown in green. The PLP cofactor covalently bound to Lys34 is shown as a black ball-and-stick model. This figure was produced in *PyMOL* (DeLano, 2002 ▶).

The tertiary structure of *A. baumannii* alanine racemase (Alr*_Aba_*) is a homodimer in which the two monomers interact in a head-to-tail manner. This results in two active sites per enzyme, each comprised of residues from the N-terminal domain of one monomer and the C-terminal domain of the second monomer (Fig. 1 ▶ *a*). Each Alr*_Aba_* monomer contains two distinct domains (Fig. 1 ▶ *b*). The N-terminal domain corresponds to residues 1–230 in the structure and predominantly consists of an eight-stranded α/β-barrel. The C-terminal domain corresponds to residues 231–356 in the structure and mainly contains β-strands (two α-helices and eight β-strands). The individual Alr*_Aba_* monomers are crystallographically distinct and form a dimer in the asymmetric unit. Following refinement, they have a low r.m.s difference of 0.41 Å after C^α^-atom superposition. Overall, the Alr*_Aba_* structure is lacking clear density for residues 247–263 and 355–356 of monomer *B*. Missing density for these regions has previously been observed in alanine racemases from other species, including *M. tuberculosis* (LeMagueres *et al.*, 2005 ▶), *B. henselae* (Abendroth *et al.*, 2011 ▶), *S. aureus* (Scaletti *et al.*, 2012 ▶) and *O. oeni* (Palani *et al.*, 2013 ▶).

**Figure 2:**
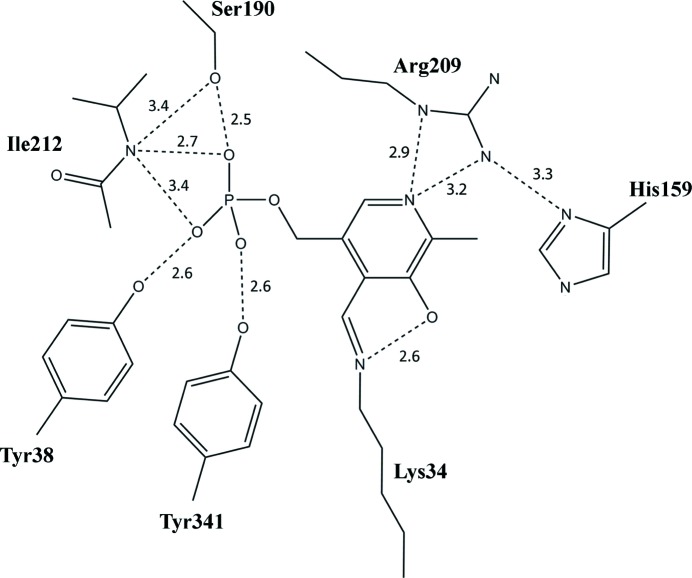
Active site of *A. baumannii* Alr, depicting the network of hydrogen bonds securing the PLP cofactor as found in monomer *A*. Hydrogen bonds are indicated by dashed lines. Bond distances are indicated in Å. Functional groups of amino acids are shown. This figure was produced using *ChemDraw Pro* (v.12.0.2).

The essential PLP cofactor is covalently bound to Lys34 *via* an internal aldimine linkage and extends towards the centre of the α/β-barrel. The pyridine N_1_ of the PLP ring is stabilized by hydrogen bonding to Arg209, which is further stabilized by interactions with His159. The phosphate tail of PLP is stabilized by several residues from one monomer. The OP_1_ of the phosphate group hydrogen bonds to Ile212 and Tyr38, OP_2_ hydrogen bonds to Try341 and OP_3_ hydrogen bonds to Ile212 and Ser190 (Fig. 2 ▶). Arg132 usually interacts with the phenolic O atom (O3′) of PLP, aiding in maintaining the position of the cofactor (Shaw *et al.*, 1997 ▶). However, Arg132 was not within hydrogen-bonding distance of PLP in the Alr*_Aba_* structure. There was no additional density in the Alr*_Aba_* structure consistent with the presence of any additional ligands besides PLP.

### 3.2. Structural and biochemical comparison with closely related alanine racemases   

#### 3.2.1. Overall topology, individual domains and inter-monomer hinge angle   

**Figure 3:**
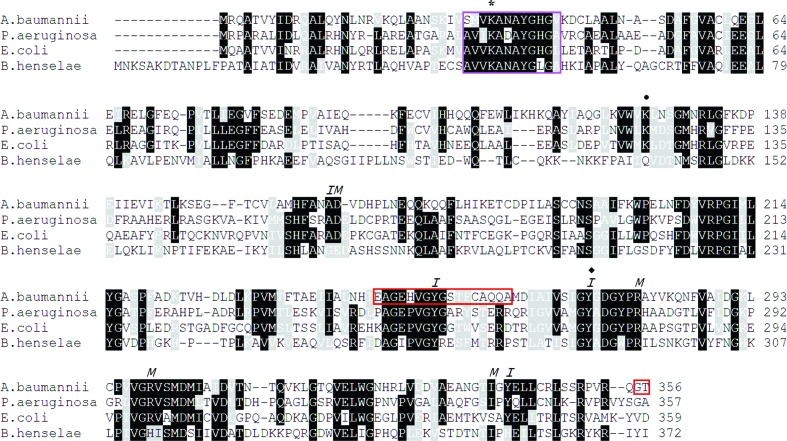
Structure-based sequence alignment of alanine racemases from *A. baumannii*, *E. coli*, *B. henselae* and *P. aeruginosa*. Identical residues are shaded black, while grey shading indicates amino acids with conserved physicochemical properties. An asterisk marks the highly conserved PLP-bound lysine and a black diamond marks the location of the catalytic tyrosine, while a black circle indicates the location of a residue which is often carbamylated in alanine racemases that have a lysine at this position. The purple box encloses the conserved PLP-binding motif containing the catalytic lysine. The red box indicates the region of electron density missing from monomer *B* of Alr*_Aba_*. *I* and *M* represent residues which form the inner and middle layers of the active-site entryway.

The multiple sequence alignment presented in Fig. 3 ▶ indicates a high level of sequence similarity between Alr*_Aba_* and the closely related alanine racemases from *P. aeruginosa* (DadX*_Pao_*), *B. henselae* (Alr*_Bhe_*) and *E. coli* (Alr*_Eco_*). The PLP-binding motif containing the catalytic Lys34 (sequence SMVKANAYGHG) is largely conserved between the various enzymes. In addition, residues which contribute to the inner and middle layers of the active-site entryway of the enzymes are also strongly conserved (Fig. 3 ▶). As noted previously, Alr*_Aba_* lacks electron density in the region containing Tyr254 (in monomer *B* only), which previous structural and kinetic studies have indicated to be part of the inner layer of the active-site entryway and the second catalytic residue involved in the reaction mechanism (Shaw *et al.*, 1997 ▶; Spies & Toney, 2003 ▶).

Superposition of the C^α^ atoms of the Alr*_Aba_* monomer with alanine racemases from Gram-negative bacteria (DadX*_Pao_*, Alr*_Bhe_* and Alr*_Eco_*) indicates a high level of structural similarity between the enzymes (Table 2 ▶). The Alr*_Aba_* monomer is most similar to Alr*_Eco_* and DadX*_Pao_*, with which it shares the lowest r.m.s differences (1.30 Å) and the highest sequence similarity (41%). Alr*_Aba_* is less similar to Alr*_Bhe_*, with which it has a higher r.m.s difference (1.86 Å) and a lower sequence identity (29%). Comparison of the individual domains and active site of Alr*_Aba_* with the other alanine racemases also indicates high structural similarity (Table 2 ▶).

**Figure 4:**
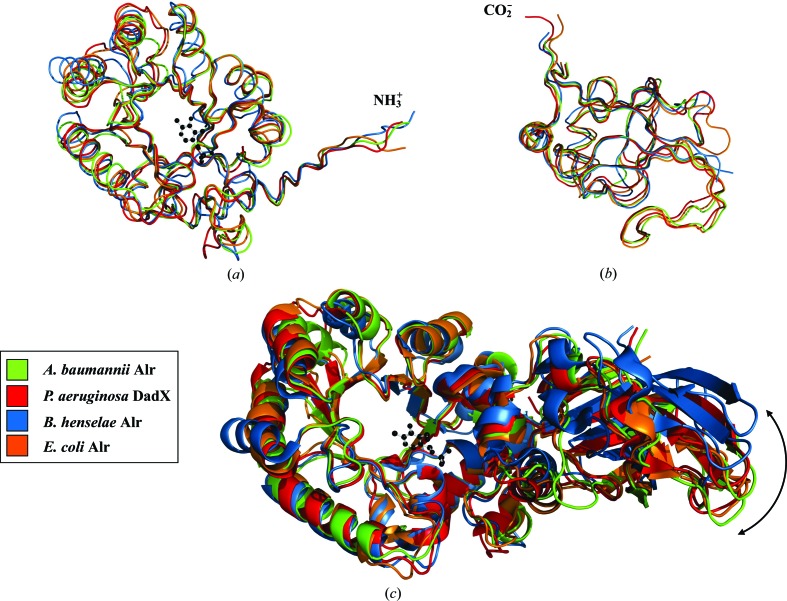
C^α^-atom superposition of Alr*_Aba_* and other alanine racemases. Colouring is as follows: *A. baumannii*, green; *P. aeruginosa*, red; *B. henselae*, blue; *E. coli*, orange. C^α^-­atom traces showing superposition between the (*a*) N-terminal and (*b*) C-terminal domains. (*c*) Superposition of the N-terminal α/β-barrel domain of whole alanine racemase monomers visualized as a ribbon representation. The PLP cofactor of Alr*_Aba_* is depicted as a black ball-and-stick model. The difference in inter-monomer hinge angle between the enzymes is indicated by a black double-headed arrow. This figure was produced in *PyMOL* (DeLano, 2002 ▶).

The N-terminal domain of Alr*_Aba_* superimposed best with those from Alr*_Eco_* and DadX*_Pao_*, with which it shares the highest sequence identity (40 and 39%, respectively) and the lowest r.m.s differences (1.32 and 1.30 Å, respectively). Consistent with the superpositions involving whole monomers, the N-terminal domain was less similar to that of Alr*_Bhe_* (Fig. 4 ▶ *a*). In the C-terminal domain, Alr*_Aba_* superimposed equally well with Alr*_Bhe_*, Alr*_Eco_* and DadX*_Pao_* (1.07, 1.02 and 1.08 Å, respectively; Fig. 4 ▶ *b*). Analysis of the active sites indicates that this region superimposed the best, having much lower r.m.s differences than whole monomers or individual domains (Alr*_Bhe_*, 0.91 Å; Alr*_Eco_*, 0.65 Å; DadX*_Pao_*, 0.56 Å). This indicates that the active site is highly conserved between the alanine racemase structures in spite of structural deviations between their domains.

Previous studies of alanine racemases have indicated that the angle between the N-terminal and C-terminal domains of the individual monomers (the inter-monomer hinge angle) differs between enzymes (LeMagueres *et al.*, 2003 ▶; Couñago *et al.*, 2009 ▶). The difference in domain orientations is the reason that individual monomers cannot be optimally superimposed as a whole (LeMagueres *et al.*, 2005 ▶; Im *et al.*, 2011 ▶). Alr*_Aba_* has a hinge angle of 141.6°, identical to that of DadX*_Pao_* (141.6°) and very similar to that of Alr*_Eco_* (140.4°), to which it has the most similar active-site architecture (Table 2 ▶). The hinge angle of Alr*_Aba_* is least similar to that of Alr*_Bhe_* (132.5°), to which it has the lowest percentage sequence identity. This indicates a positive correlation between sequence identity and inter-monomer hinge angle. To date, the best explanation for hinge-angle differences involves the hydrogen-bond interactions formed between residues from the N- and C-terminal tails of opposite monomers (LeMagueres *et al.*, 2003 ▶, 2005 ▶; Couñago *et al.*, 2009 ▶). Alr*_Bhe_* and alanine racemases from *G. stearothermophilus* (Shaw *et al.*, 1997 ▶), *B. anthracis* (Couñago *et al.*, 2009 ▶) and *S. aureus* (Scaletti *et al.*, 2012 ▶) have additional residues in these areas capable of forming these hydrogen bonds, resulting in similar hinge angles. Shorter alanine racemases such as Alr*_Eco_*, DadX*_Pao_* and Alr*_Aba_* do not have these additional residues (Fig. 3 ▶), resulting in differing hinge angles.

#### 3.2.2. Enzyme kinetics   

Kinetic characterization of Alr*_Aba_* revealed that the enzyme has a *V*_max_ of 11.3 U mg^−1^ and a *K*_m_ of 0.56 m*M* for the racemization of d-alanine to l-alanine (Table 3 ▶). The other direction of the racemization was not characterized in this study. This is very similar to the kinetic values reported for Alr*_Eco_* (*V*_max_ = 8.8 U mg^−1^, *K*_m_ = 0.31 m*M*), with which it shares high structural similarity (Table 2 ▶) and a similar hinge angle. However, despite Alr*_Aba_* sharing a high structural similarity and hinge angle with DadX*_Pao_*, the kinetic parameters differed significantly between these enzymes (*V*_max_ = 134 U mg^−1^ and *K*_m_ = 1.40 m*M* for DadX*_Pao_*). This indicates that despite high levels of sequence identity, similar active-site structures and hinge angles, the kinetics parameters of even closely related alanine racemases can differ. This is in agreement with what has been observed in previous studies (Couñago *et al.*, 2009 ▶; Scaletti *et al.*, 2012 ▶). No comparison could be made with Alr*_Bhe_* as no kinetic information is available.

#### 3.2.3. Dimer interface, substrate entryway and active site   

Previous studies have found that alanine racemase is dependent on dimerization for enzyme activity, and that a number of residues involved in the dimer interface are highly conserved (Strych & Benedik, 2002 ▶; Im *et al.*, 2011 ▶; Scaletti *et al.*, 2012 ▶). This makes the dimer interface a potential target for structure-aided drug design. The area of the dimer interface of Alr*_Aba_* is 2360 Å^2^ and is formed by roughly equal contributions from both monomers (73 and 70 residues from monomer *A* and monomer *B*, respectively). This interface contains five salt bridges and 22 hydrogen-bond interactions. The dimer interface of Alr*_Aba_* is most similar to the interface calculated for Alr*_Bhe_* (2606 Å^2^), which has similar numbers of residues involved in dimerization (71 and 75 in monomers *A* and *B*, respectively). The interface is less similar to the interfaces calculated for DadX*_Pao_* (1918 Å^2^) and Alr*_Eco_* (2816 Å^2^), both of which have a larger number of residues involved in dimerization (DadX*_Pao_*, 82 and 82 in monomers *A* and *B*, respectively; Alr*_Eco_*, 81 and 83 in monomers *A* and *B*, respectively).

The substrate entryway of Alr*_Aba_* is comprised of residues from both monomers and consists of an outer, a middle and an inner layer (with the inner layer being in closest proximity to the PLP cofactor and the outer layer being on the surface of the protein). Comparison of Alr*_Aba_* with the other enzymes indicates that the inner (Ala163, Tyr254′, Tyr273′ and Tyr341) and middle (Asp164, Arg279, Arg299 and Ile339) layers of the entryway are highly conserved (Fig. 3 ▶), consistent with previous studies (LeMagueres *et al.*, 2005 ▶; Im *et al.*, 2011 ▶). The highly conserved nature of the alanine racemase substrate entryway makes it a potential target for the development of enzyme-specific inhibitors.

**Figure 5:**
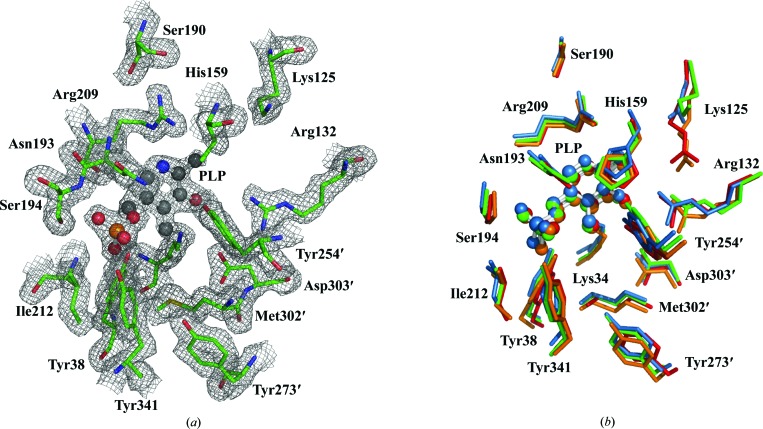
Active site of *A. baumannii* alanine racemase. (*a*) 2*F*_o_− *F*_c_ electron-density map of the active site contoured at 1.0σ with isomesh map shown (1.6 Å carve). The main-chain and side-chain atoms of the Alr*_Aba_* active-site residues are depicted as sticks. C atoms are green, O atoms red, N atoms blue, S atoms yellow and phosphates orange. The PLP cofactor is depicted as a ball-and-stick model in which C atoms are coloured black. (*b*) Superposition of the active-site residues of alanine racemases from *A. baumannii* (green), *B. henselae* (blue), *P. aeruginosa* (red) and *E. coli* (orange). For *A. baumannii*, a hybrid view is depicted with residues from monomer *B*, except for the side chain of His159 which is included from monomer *A*. The PLP cofactors from each structure are depicted as ball-and-stick models. Primes denote residues contributed by the second monomer. The superposition was performed using the residue ranges stated in Table 2 ▶. This figure was produced in *PyMOL* (DeLano, 2002 ▶).

The active-site residues of Alr*_Aba_* and other alanine racemases superimpose particularly well, with low r.m.s differences (Table 2 ▶). The active-site structure of Alr*_Aba_* is most similar to that of DadX*_Pao_* (0.56 Å; 58% sequence identity). Alr*_Aba_* also shares high similarity to Alr*_Eco_* (0.65 Å; 60% sequence identity), but diverges most from Alr*_Bhe_* (0.91 Å; 48% sequence identity). This indicates a positive correlation between sequence identity and structural similarity in this region. As mentioned in §[Sec sec3.1]3.1, the active site of Alr*_Aba_* is composed of residues from both monomers, several of which are involved in a hydrogen-bond network with the PLP cofactor (Figs. 2 ▶ and 5 ▶ *a*) and are conserved between alanine racemases (Fig. 5 ▶ *b*).

Alr*_Aba_* is lacking density for Tyr254′ in one active site and previous studies have shown that this residue is important in substrate binding and catalysis (Shaw *et al.*, 1997 ▶; Morollo *et al.*, 1999 ▶; Watanabe *et al.*, 2002 ▶). As noted above, missing density for this residue has previously been observed in the active sites of several alanine racemases (LeMagueres *et al.*, 2005 ▶; Abendroth *et al.*, 2011 ▶; Scaletti *et al.*, 2012 ▶; Palani *et al.*, 2013 ▶). His159 can be identified in the same active site and its side chain forms a hydrogen bond to Arg209, as well as a parallel displaced π-stacking interaction with the PLP ring. The closest contact distance for this interaction is ∼3 Å, which is consistent with other reports in proteins (McGaughey *et al.*, 1998 ▶).

In the second active site Tyr254′ is present but the His159 side chain is difficult to localize, with little density beyond the β-carbon. A hybrid view with these residues included from opposite active sites (Fig. 5 ▶ *b*) reveals that these two residues are located in their usual positions when compared with the active sites from other bacterial alanine racemase structures.

Differences between the Alr*_Aba_* active site and the other alanine racemase structures included a lack of density consistent with Lys125 carbamylation (Figs. 3 ▶ and 5 ▶ *b*). This modification is present in both Alr*_Eco_* and DadX*_Pao_*; it is proposed to aid in the positioning of Arg132 within the active site (Morollo *et al.*, 1999 ▶; LeMagueres *et al.*, 2003 ▶; Wu *et al.*, 2008 ▶) and is a process that is dependent on a relatively high pH (>8.0; Golemi *et al.*, 2001 ▶). It is possible that the observed lack of carbamylation in the Alr*_Aba_* structure is owing to the low pH (6.0) at which the enzyme was crystallized. Furthermore, Alr*_Aba_* did not contain any extra density consistent with other molecules in its active site. Previous alanine racemase structures have reported the presence of additional molecules in the active site such as sulfate (Scaletti *et al.*, 2012 ▶), chloride (Couñago *et al.*, 2009 ▶) and acetate (Shaw *et al.*, 1997 ▶; Couñago *et al.*, 2009 ▶; Scaletti *et al.*, 2012 ▶). The lack of substrate-like molecules in the active site could also be a contributory factor to the unusual position of Arg132 owing to this residue playing an integral role in substrate binding.

## 4. Conclusions   

We have reported the crystal structure of *A. baumannii* alanine racemase from the highly antibiotic-resistant NCTC13302 strain to 1.9 Å resolution. The overall structure is very similar to closely related alanine racemases from Gram-negative bacteria. Interestingly, the partial kinetic analysis generated in this study indicated an inconsistent relationship between structural similarity and racemization rate. The substrate entryway and active sites of these alanine racemases were shown to be highly conserved and are thus possible targets for the development of enzyme-specific inhibitors. The structural determination and biochemical analysis presented here could provide a template for future structure-based drug-design studies targeting Alr*_Aba_*.

## Supplementary Material

PDB reference: alanine racemase, 4tlo

## Figures and Tables

**Table 1:** Data-collection and refinement statistics Values in parentheses are for the highest resolution shell.

Space group	*P*2_1_
Unit-cell parameters	
*a* (Å)	47.0
*b* (Å)	83.0
*c* (Å)	93.3
β (°)	97.1
No. of observations	406799 (28844)
No. of unique reflections	55904 (3960)
Completeness (%)	99.7 (99.7)
*R*_merge_ † (%)	9.2 (26.0)
〈*I*/σ(*I*)〉	16.9 (8.4)
Multiplicity	7.3 (7.3)
Resolution range (Å)	30.15–1.90 (1.95–1.90)
*R* factor‡ (%)	19.7
*R*_free_(%)	23.4
Average *B* factors (Å^2^)	
All atoms	14.8
Main chain	13.3
Side chains	15.3
Waters	20.5
R.m.s. deviations	
Bond lengths (Å)	0.016
Bond angles (°)	1.75
No. of atoms	
Protein	5360
PLP	30
Water	512

† *R*_merge_ = 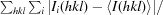 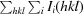.

‡ *R* factor = 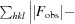 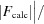 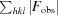.

**Table 2:** Average r.m.s differences (Å) between the C^α^ atoms of Alr*_Aba_* and other alanine racemases Numbers in parentheses denote sequence identity with Alr*_Aba_*. Residues from the other structures equivalent to those in Alr*_Aba_* monomer *A* were used for the superpositions.

Alanine racemase	PDB entry	Whole monomer†	N-terminal domain‡	C-terminal domain§	Active site¶
Alr*_Eco_*	2rjg	1.30 (41%)	1.32 (40%)	1.02 (43%)	0.65 (60%)
Alr*_Bhe_*	3kw3	1.86 (29%)	1.68 (25%)	1.07 (36%)	0.91 (48%)
DadX*_Pao_*	1rcq	1.30 (41%)	1.30 (39%)	1.08 (45%)	0.56 (58%)

† Calculated using monomer *A* for Alr*_Eco_* and DadX*_Pao_* and monomer *B* for Alr*_Bhe_*.

‡ Calculated using residues 1–230.

§ Calculated using residues 231–354.

¶ Calculated using residues 32–38, 54–58, 75–79, 95–99, 121–134, 154–162, 188–195, 206–213 and 338–345 from monomer *B* and 252–255, 272–276 and 299–304 from monomer *A* for Alr*_Eco_* and DadX*_Pao_* and *vice versa* for Alr*_Bhe_*.

**Table 3:** Kinetic parameters for the racemization between L- and D-alanine by alanine racemases NR, value not reported.

	L-to-D direction		D-to-L direction	
Alanine racemase	*K*_m_ (m*M*)	*V*_max_ † (U mg^−1^)	*K*_m_ (m*M*)	*V*_max_ † (U mg^−1^)
Alr*_Aba_* ‡	NR	NR	0.56§	11.3§
Alr*_Eco_* [Table-fn tfn10]	1.0§	356§	0.31§	8.8§
Alr*_Bhe_*	NR	NR	NR	NR
DadX*_Pao_* [Table-fn tfn11]	1.40[Table-fn tfn12]	155[Table-fn tfn12]	1.40[Table-fn tfn12]	134[Table-fn tfn12]

† One unit is defined as the amount of enzyme which catalyzes the racemization of 1 µmol of substrate per minute.

‡ Kinetic parameters reported in the current work.

§ Assay performed at 30°C.

¶ Kinetic parameters reported by Wu *et al.* (2008 ▶).

†† Kinetic parameters reported by Strych *et al.* (2000 ▶).

‡‡ Assay performed at 23°C.
